# Soluble programmed cell death receptor-1 (sPD-1): a potential biomarker with anti-inflammatory properties in human and experimental acute respiratory distress syndrome (ARDS)

**DOI:** 10.1186/s12967-016-1071-x

**Published:** 2016-11-11

**Authors:** Sean F. Monaghan, Chun-Shiang Chung, Yaping Chen, Joanne Lomas-Neira, William G. Fairbrother, Daithi S. Heffernan, William G. Cioffi, Alfred Ayala

**Affiliations:** 1 Division of Surgical Research, Department of Surgery, Alpert School of Medicine at Brown University and Rhode Island Hospital, 593 Eddy Street, Providence, RI 02903 USA; 2 MCB Department, Brown University, 70 Ship Street, Providence, RI 02903 USA

**Keywords:** Acute respiratory distress syndrome, ARDS, Soluble PD-1, PD-1, Biomarker, Immunotherapy

## Abstract

**Background:**

Acute respiratory distress syndrome (ARDS) remains a common organ dysfunction in the critically ill patient. Mechanisms for its development have focused on immune mediated causes, aspects of our understanding are not complete, and we lack biomarkers.

**Design, setting, and subjects:**

Blood and bronchial alveolar lavage fluid (BAL) from humans (n = 10–13) with ARDS and controls (n = 5–10) as well as a murine model of ARDS (n = 5–6) with controls (n = 6–7) were studied.

**Methods:**

ARDS was induced in mice by hemorrhagic shock (day 1) followed by poly-microbial sepsis (day 2). Samples were then collected on the third day after the animals were euthanized. Ex vivo experiments used splenocytes from animals with ARDS cultured with and without soluble programmed death receptor-1 (sPD-1).

**Results:**

Levels of sPD-1 are increased in both the serum (11,429.3 pg/mL(SD 2133.3) vs. 8061.4(SD 4187.8), p = 0.036) and bronchial alveolar lavage (BAL) fluid (6,311.1 pg/mL(SD 3758.0) vs. 90.7 pg/mL(SD 202.8), p = 0.002) of humans with ARDS. Similar results are seen in the serum (9396.1 pg/mL(SD 1546.0) vs. 3464.5 pg/mL(SD 2511.8), p = 0.001) and BAL fluid (2891.7 pg/mL(SD 868.1) vs. 1385.9 pg/mL(SD 927.8), p = 0.012) of mice. sPD-1 levels in murine blood (AUC = 1(1–1), p = 0.006), murine BAL fluid (AUC = 0.905(0.717–1.093), p = 0.015), and human BAL (AUC = 1(1–1), p = 0.001) fluid predicted ARDS. To assess the importance of sPD-1 in ARDS, ex vivo experiments were undertaken. BAL fluid from mice with ARDS dampens the TNF-α production compared to cells cultured with BAL lacking sPD-1 (2.7 pg/mL(SD 3.8) vs. 52.38 pg/mL(SD 25.1), p = 0.002).

**Conclusions:**

This suggests sPD-1 is elevated in critical illness and may represent a potential biomarker for ARDS. In addition, sPD-1 has an anti-inflammatory mechanism in conditions of marked stress and aids in the resolution of severe inflammation. sPD-1 could be used to not only diagnose ARDS, but may be a potential therapy.

## Background

Acute respiratory distress syndrome (ARDS) is seen in critically ill patients [1] affecting 190,000 patients each year in the United States [2]. The mechanisms of ARDS are not fully understood but the immune system is implicated. Neutrophils and the epithelial/endothelial cell border dysfunction in the lung are potential contributors to ARDS pathophysiology [2, 3].

Programmed death receptor-1 (PD-1) is an immune modulating/co-inhibitory cell-surface protein involved in chronic conditions such as cancer, autoimmune diseases, and viral infection [4, 5]. Recently, acute conditions like sepsis and ARDS have been influenced by the expression of PD-1. In both humans and mice, PD-1 appears to play a role in the pathology as well as outcomes from sepsis [6–8] and ARDS [9]. In sepsis the influence of PD-1 appears to be modulated through expression on macrophages as survival is improved in animals lacking PD-1 [8]. In addition animals in which PD-1 antibody is administered have enhanced survival and clinical trials for PD-1 in sepsis are planned [6]. The survival benefit of mice lacking PD-1 with ARDS appears to be due to alterations in cell ratio, TNF expression and neutrophil function [9].

PD-1 mediates its inhibitory action(s) via cell-surface ligands. There are reports of a soluble form of PD-1 (sPD-1) due to alternative splicing [10] with levels present in patients/experimental animals with autoimmune diseases [11], chronic viral infection [12], and cancer [13].

Alternative splicing is known to occur in up to 90% of genes with multiple exons [14]. Factors that influence alternative splicing (acidosis, hypoxia) [15–17] are also prominent in critical illness. The role of alternative splicing and sPD-1 has not been characterized in acute conditions like ARDS. Here we reveal an association between sPD-1 and ARDS across two species (human and mouse) and propose a novel mechanism for sPD-1.

## Methods

### Collection of human samples

Samples were obtained from patients in trauma and surgical intensive care units undergoing routine analysis of their blood or a bronchial alveolar lavage (BAL) for suspected pneumonia. Patients were diagnosed with ARDS based on the definition provided by Bernard and colleagues [18] and consistent with current criteria [1]. Controls for the serum analysis came from ventilated, critically ill patients who did not have ARDS. Controls for BAL samples were obtained from outpatients undergoing an elective bronchoscopy. The BAL samples were always the first washing with saline (typically discarded). BAL fluid was centrifuged and the supernatant was taken to ensure no cells were in the samples. The Institutional Review Board of Rhode Island Hospital approved this study and waived documentation of consent (protocol number 211087).

### Induction of indirect ARDS in mice

C57BL/6 male mice (The Jackson Laboratory, Bar Harbor, ME) between 10 and 12 weeks were used. Experiments were done according to guidelines from the National Institutes of Health (Bethesda, MD) and were approved by the Rhode Island Hospital animal use committee (number 0050-08). Isoflurane was used for anesthesia and euthanasia was done by CO_2_ overdose followed by cardiac puncture. ARDS was induced in the mice by hemorrhage (non-lethal shock) followed by cecal ligation and puncture (CLP) [9, 19–22]. The control group was sham hemorrhage followed by sham CLP (Sham–Sham). The same aged and gender mice were used in all experiments, including those utilizing ex vivo splenocytes.

### Measurement of PD-1 and sPD-1

Blood from mice and humans was centrifuged at 10,000×*g* at 4°C for 10 min, the serum layer was isolated, and red blood cells were lysed with 1 mL double distilled water with 0.037 g EDTA (Invitrogen, Carlsbad, CA), 8.26 g NH_4_Cl (Sigma, St. Louis, MO), and 1 g KHCO_3_ (Sigma, St. Louis, MO). Using the BD FACSArray, human lymphocytes were phenotyped for expression of PD-1 with PE labeled anti-PD-1 (Ms IgG1, Biolegend) and APC anti-CD3 (UCHT1, Beckman Coulter). In mice, PE labeled anti-PD-1 (J43, eBioscience) and APC labeled anti-CD3 (145-2C11, eBioscience) were used.

sPD-1 levels were assessed by ELISA. Microtiter plates were coated overnight with the 200 μL of sample (BAL fluid or serum) or a serial dilution of concentrations of the PD-1 Fc Chimera (R&D Systems) to serve as the controls. Wells were blocked and washed per standard ELISA protocol (R&D Systems). Anti-PD-1 antibody (NB110-86970, Novus Biologicals) was used for detection and after incubation for 2 h, anti-rabbit IgG HRP (GE Health Care Systems) was added, incubated for 1 h and plates were washed. TMB substrate solution was then added and incubated in the dark for 5 min. A stop solution (2 NH_2_SO_4_) was added and the optical density was determined at 450 nm in a microplate reader.

In order to ensure that the detected sPD-1 was the result of alternative splicing and not the detection of the membrane bound form in apoptotic bodies, the RNA expression was assessed. The RNA was isolated from cell pellets from the peripheral blood of humans in the intensive care unit with and without ARDS. The RNA was isolated as previously described [23]. Full length (fl) PD-1 represents PD-1 on the cell surface while PD-1ex3 correlates to sPD-1. GAPDH was used as a house-keeping gene and results are displayed as fold expression relative to it.

The following primers were used for flPD-1, 5′-TCAGGGTGACAGAGAGAAG-3′ and 5′-GACACCAACCACCAGGGTTT-3′; GAPDH, 5′-GTGAAGGTCGGAGTCAACG-3′ and 5′-TGAGGTCAATGAAGGGGTC-3′; PD-1ex3, 5′-AGGGTGACAGGGACAATAGG-3′ and 5′-CCATAGTCCACAGAGAACAC-3′.

### Ex vivo experiments

Mice either underwent no surgical intervention (naïve), hemorrhage and CLP as above (ARDS), or underwent sham hemorrhage and sham CLP (control). Mice were male and between 10 and 12 weeks of age. Spleens from mice were harvested after the mice were euthanized. The spleens were crushed between two slides to liberate the cells into 10 mL sterile PBS. Red blood cells were lysed with sodium chloride. The solution was centrifuged down and cells were counted. Splenocytes were cultured with DMEM (Invitrogen) with 10% fetal calf serum (Atlantic Biologicals) and 0.1% gentamycin (Sigma).

BAL fluid from mice with ARDS (see above) was then added to the cultured splenocytes. In order to decipher the impact of sPD-1 in the BAL fluid, 5 μg of anti-PD-1 antibody (NB110-86970, Novus Biologicals) per 50 ug of target protein was added and incubated overnight at 4 °C. 200 μL of Protein A/G (Santa Cruz was added and incubated for 2 h at 4 °C. The fluid was then centrifuged at 12,000 rpm for 15 min and the supernatant collected. Naïve splenocytes from five different mice were cultured with 50 μL of BAL fluid from mice with ARDS per 100,000 cells in each of the above three groups.

Differing levels of a recombinant PD-1 Fc Chimera (R&D Systems) or IgG (R&D Systems) control was then used as an additive to the culture of splenocytes (as above) from mice with ARDS compared to mice who underwent sham hemorrhage and sham CLP as previously described [12, 13].

All cells were cultured for 72 h. At that point, proliferation was assessed using the CyQuant assay (Invitrogen). Supernatants were collected and analyzed for the production of IL-6, IL-10, MCP-1, IFN-γ, TNF-α, and IL-12 using the commercially available BD Cytometric Bead Array mouse inflammation kit (BD Biosciences, Franklin Lakes, NJ). In order to assess if apoptosis and subsequent apoptotic bodies were influencing the results of the above experiments, splenocytes were cultured with 0, 1000 and 10,000 pg/mL of the fusion protein for 3 days and apoptosis was assessed using flow cytometry assessment of Annexin V (PE labeled).

Data was analyzed using SigmaPlot 10.0 (Systat Software, San Jose, CA). Paired t tests or rank sum analysis were done when two groups are compared. Multiple groups were compared by one-way or two-way ANOVA with Student-Newman-Keuls correction. Graphs are displayed as mean or median with error bars representing the standard deviation. Alpha was set to 0.05.

## Results

### PD-1 and sPD-1 expression in ARDS

**Fig. 1:**
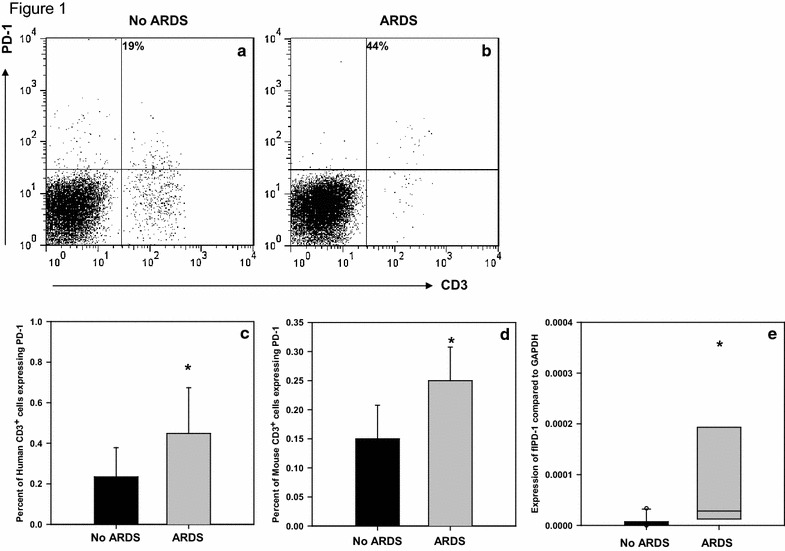
PD-1 Expression on CD3+ T-cells in the blood of humans and mice with ARDS. **a** Representative data showing staining and gating of human sample without ARDS. **b** Representative data showing staining and gating of human sample with ARDS. **c** Percent of CD3+ T-cells in the blood of humans expressing PD-1 in patients with ARDS (n = 21) compared to those without ARDS (n = 9). Analysis done by t test, *p = 0.014 when compared to patients without ARDS. **d** Percent of CD3^+^ T-cells in the blood expressing PD-1 in mice with ARDS (n = 4) compared to those without ARDS (n = 4). Analysis done by t test, *p = 0.05 when compared to patients without ARDS. **e** flPD-1 expression in folds compared to GAPDH expression in patients with and without ARDS. Analysis done by rank sum test. *p = 0.004 when compared to patients without ARDS

The expression of PD-1 on lymphocytes was compared in critically ill patients with ARDS (n = 21) to other ICU patients without ARDS (n = 9). The groups had similar gender distribution (38% female vs. 55%, p = 0.443) and APACHE II scores (22.0 vs. 19.6, p = 0.331). PaO_2_:FiO_2_ ratio was significantly decreased in patients with ARDS (207.9 vs. 446.0, p < 0.001). The levels of PD-1 expression on CD3+ T-cells were significantly increased in patients with ARDS (Fig. 1c). Similar results were seen in the expression of PD-1 on CD3+ T-cells in mice with ARDS [19] compared to controls (Fig. 1d). In addition, blood samples from patients with ARDS had increased expression of full length PD-1 compared to ICU patients without ARDS (Fig. 1e).

**Fig. 2:**
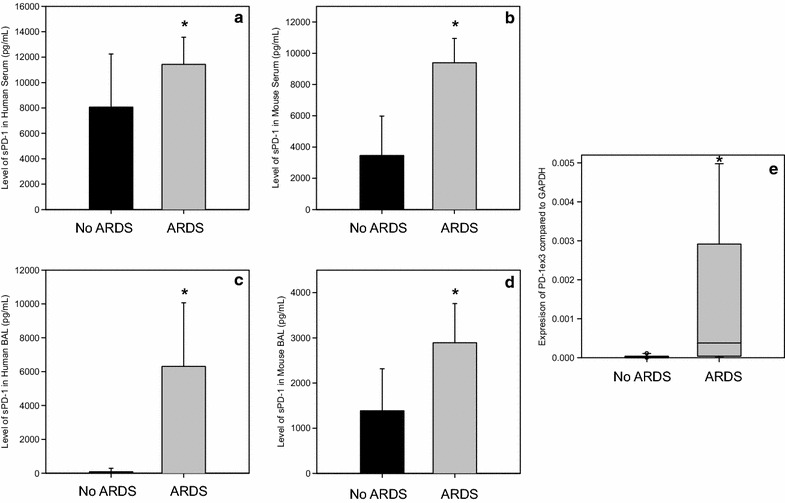
Levels of soluble PD-1 in the serum and bronchial alveolar lavage fluid of humans and mice with and without ARDS measured by ELISA. **a** sPD-1 levels in human serum in patients with ARDS (n = 10) compared to those without ARDS (n = 10). Analysis done by t test, *p = 0.036 when compared to patients without ARDS. **b** sPD-1 levels in mouse serum in mice with ARDS (n = 5) compared to those without ARDS (n = 6). Analysis done by t test, *p = 0.001 when compared to mice without ARDS. **c** sPD-1 levels in human bronchial alveolar lavage fluid in patients with ARDS (n = 13) compared to those without ARDS (n = 5). Analysis done by t test, *p = 0.002 when compared to patients without ARDS. **d** sPD-1 levels in mouse bronchial alveolar lavage fluid in mice with ARDS (n = 6) compared to those without ARDS (n = 7). Analysis done by t test, *p = 0.012 when compared to mice without ARDS. **e** PD-1ex3 expression in folds compared to GAPDH expression in patients with and without ARDS. Analysis done by rank sum test. *p = 0.002 when compared to patients without ARDS

The systemic levels of sPD-1 were measured in ARDS patients and were compared to controls. The patients with ARDS (n = 10) had similar APACHE II scores (21.8 vs. 21.1 p = 0.778), age (61.5 vs. 59.8, p = 0.835) and gender distribution (50% female vs. 50%, p = 0.655) but significantly different PaO_2_:FiO_2_ ratio (209.3 vs. 446.2 p < 0.001) when compared to ICU patients without ARDS (n = 10) (please see Table 1). The level of sPD-1 in the serum was significantly higher in patients with ARDS (11,429.3 pg/mL vs. 8061.4, p = 0.036) (Fig. 2a). This data was supported by peripheral blood leukocyte gene expression results showing that in ARDS patients there was increased expression of PD-1∆Ex3 (mRNA for sPD-1) compared to controls (Fig. 2e). In mice, a similar rise in the serum levels of sPD-1 was seen in those animals with ARDS (9396.1 vs. 3464.5 pg/mL, p = 0.001, Fig. 2b).

**Table 1:** Demographic and clinical data of human samples

Demographics of patients whose serum sPD-1 was assessed			
	ARDS (n = 10)	No ARDS (n = 10)	p value
Age	62	60	0.835
Female	50%	50%	0.655
APACHE II	22	21	0.778
PaPO_2_:FiO_2_	209	357	<0.001
sPD-1 level	11,429 pg/mL	8061 pg/mL	0.036

Demographics of patients whose BAL fluid sPD-1 was assessed			
	ARDS (n = 13)	No ARDS (n = 5)	p value
Age	58	62	0.678
Female	58%	62%	0.596
sPD-1 level	6311 pg/mL	90.7 pg/mL	0.002

BAL fluid showed the levels of sPD-1 to be significantly higher in patients with ARDS (6311.1 vs. 90.7 pg/mL, p = 0.002; n = 13) when compared to controls (n = 5) (Fig. 2c). Age (58 vs. 62.2, p = 0.678) and gender distribution (53.8% female vs. 46.2% female, p = 0.596) were not different between patients with ARDS and controls (please see Table 1). Again, a similar result was seen as the sPD-1 levels in the BAL fluid from mice with ARDS (2891.7 pg/mL) were higher compared to control mice (1385.9 pg/mL, p = 0.012, Fig. 2d).

F curves were plotted and sPD-1 levels in murine blood (AUC = 1 (1–1), p = 0.006), murine BAL fluid (AUC = 0.905 (0.717–1.093), p = 0.015), and human BAL (AUC = 1 (1–1), p = 0.001) fluid predicted ARDS. sPD-1 levels in human serum came close to diagnostic significance (AUC 0.745 (0.526–0.964), p = 0.06).

### Potential mechanism of sPD-1’s action

**Fig. 3:**
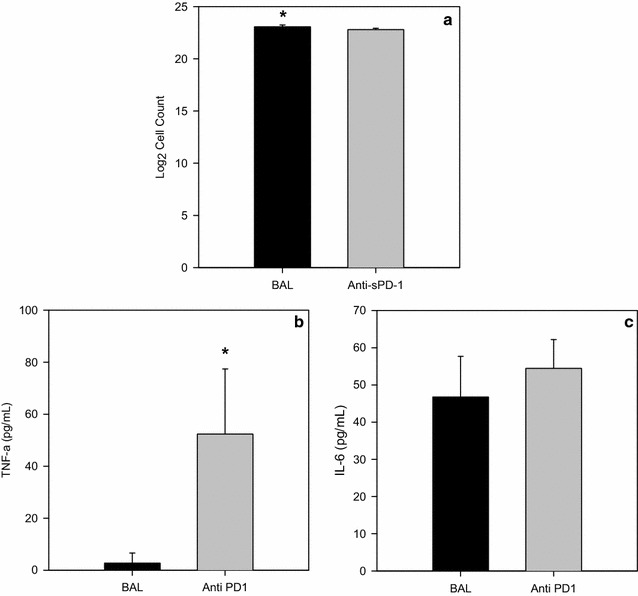
Outcomes of cells cultured with BAL fluid from mice with ARDS. **a** Proliferation of splenocytes from naïve mice cultured with BAL fluid from mice with ARDS (BAL, n = 5, experiments in triplicate) compared to cells cultured with BAL fluid where the sPD-1 was precipitated out (Anti-sPD-1, n = 5, experiments in triplicate) Analysis done by t test, *p = 0.032 when compared to cells cultured with BAL fluid where sPD-1 was precipitated out. **b** TNF-α levels of splenocytes from naïve mice cultured with BAL fluid from mice with ARDS (BAL, n = 5) compared to cells cultured with BAL fluid where the sPD-1 was precipitated out (Anti-sPD-1, n = 5) Analysis done by t test, *p = 0.002 when compared to cells cultured with BAL fluid from mice with ARDS. **c** IL-6 levels of splenocytes from naïve mice cultured with BAL fluid from mice with ARDS (BAL, n = 5) compared to cells cultured with BAL fluid where the sPD-1 was precipitated out (Anti-sPD-1, n = 5)

To assess the biological role of sPD-1 in ARDS, BAL fluid from mice with ARDS was cultured with splenocytes from naïve mice. When cells were cultured with BAL fluid from mice with ARDS, a small increase in proliferation was seen compared to the BAL fluid lacking sPD-1 (p = 0.032, Fig. 3a). Alternatively, when basal levels of cytokine production were measured between these three groups, no IL-10, IL-12, IFN- γ, or MCP-1 was detected, but cells cultured with the ARDS mouse BAL fluid had significantly less TNF-α production (2.7 pg/mL) compared to cells cultured with ARDS mouse BAL fluid in which the sPD-1 precipitated out (52.38 pg/mL, p = 0.002, Fig. 3b). Cells cultured with ARDS mouse BAL fluid had similar IL-6 levels when compared to cells exposed to ARDS mouse BAL where sPD-1 was removed (46.74 vs. 54.46, p = 0.234, Fig. 3c).

Splenocytes harvested from mice with/without ARDS may respond differently when cultured with varying levels of recombinant human sPD-1 we used a two-way analysis of variance to test the proliferative capacity of splenocytes from mice with and without ARDS when cultured with varying levels of recombinant sPD-1 (Fig. 3a). Cells from mice with ARDS showed a similar degree of proliferation to cells taken from mice that underwent sham procedures (F (1, 24) = 0.753, p = 0.394). Differences in the amount of sPD-1 the cells were cultured with resulted in significantly different proliferation (F (5, 24) = 6.32, p < 0.001). The interaction of the type of cell (cells from mice with ARDS or control mice) and the amount of sPD-1 cultured with the cells was not significant (F (5, 24) = 0.306, p = 0.904). When assessing the extent of proliferation in the presence of varying levels of sPD-1, 1000, 2500, 5000 and 10,000 pg/mL had significantly more (p < 0.05) proliferation compared to 0 and 1000,000 pg/mL of sPD-1 (Fig. 3a).

**Fig. 4:**
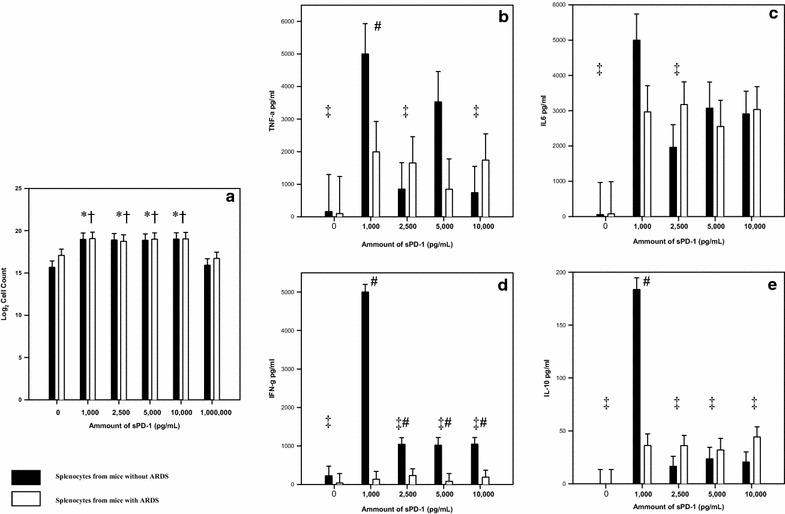
Outcome of cells cultured with varying levels of sPD-1 **a** Proliferation of splenocytes from mice with ARDS (n = 4, experiments in triplicate) compared to mice without ARDS (n = 4, experiments in triplicate). Analysis done by two-way ANOVA with Student-Newman-Keuls method used for multiple comparisons. *p < 0.05 when that amount is compared to 0 ng/mL regardless of whether the cells came from a mouse with ARDS. ^†^p < 0.05 when that amount is compared to 1000 ng/mL regardless of whether the cells came from a mouse with ARDS. **b** TNF-α levels from splenocytes of mice with ARDS (n = 4) compared to mice without ARDS (n = 4) ^#^p < 0.05 when cells from mice with ARDS are compared to cells from sham mice at that level of sPD-1 ^‡^p < 0.05 when comparing cells from sham mice to a sPD-1 level of 1 ng/mL **c** IL-6 levels from splenocytes from mice with ARDS (n = 4) compared to mice without ARDS (n = 4) ^‡^p < 0.05 when comparing cells from sham mice to a sPD-1 level of 1 ng/mL **d** IFN-γ levels from splenocytes from mice with ARDS (n = 4) compared to mice without ARDS (n = 4) ^#^p < 0.05 when cells from mice with ARDS are compared to cells from sham mice at that level of sPD-1 ^‡^p < 0.05 when comparing cells from sham mice to a sPD-1 level of 1 ng/mL **e** IL-10 levels from splenocytes from mice with ARDS (n = 4) compared to mice without ARDS (n = 4) ^#^p < 0.05 when cells from mice with ARDS are compared to cells from sham mice at that level of sPD-1 ^‡^p < 0.05 when comparing cells from sham mice to a sPD-1 level of 1 ng/mL

Culturing cells from ARDS mice with/without varying concentrations of recombinant sPD-1 led to differences in cytokine release when compared to cells derived from sham animals cultured with similar concentration of sPD-1. To ensure apoptotic bodies did not influence the findings of these ex vivo experiments, Annexin V expression was similar on cells cultured with 0, 1000 and 10,000 pg/mL of the fusion protein (0.5, 0.6, 0.7%, p 0.33). Similar apoptosis was not due to lack of proliferation (6.1 × 10^4^, 6.6 × 10^4^, 6.7 × 10^4^, p = 0.87). In cells from mice with ARDS, while a modest rise in the production of TNF-α could be seen in the cells treated with sPD-1, this increase was not statistically significant. Further the presence of increasing concentrations of sPD-1 did not alter ARDS mouse splenocyte TNF-α release (p = 0.701, Fig. 4b). Alternatively, TNF-α production by cells from Sham-control mice when cultured at 1000 pg/mL of sPD-1 rose significantly when compared to either the 0 pg sPD-1 treated cells or cells derived ARDS mice also treated with 1000 pg/mL (p = 0.033, Fig. 4b). This rise was, however, transient in nature for as recombinant sPD-1 levels were increased the production of TNF-α was markedly suppressed. With regards to IL-10, IFN-γ and IL-6 we saw essentially the same affect of sPD-1 treatment on the production of these mediators by Sham-mouse cells and the ARDS mouse cells (Fig. 4c–e).

## Discussion

Previous research on PD-1 has focused on chronic inflammatory conditions. Here we have examined the expression of PD-1 using ARDS as a model disease for an acute response. In humans and mice with ARDS, the circulating T-cells exhibit increased levels of PD-1 and its gene expression (Fig. 1). Previous research has shown that in mice with ARDS there is increased expression of PD-1 not only on T-cells but also on neutrophils and dendritic cells in the lung [9]. As such, the relative decrease in number of CD-3 lymphocytes in ARDS is not as concerning since there is a global increase (across many cell lines) in PD-1 expression in mice and humans with ARDS.

The role of sPD-1 has not been consistent among diseases associated with elevated PD-1. Increases in sPD-1 were not seen in some diseases [24] but elevations were present in arthritis [23] and aplastic anemia [11]. Similar concomitant elevations of sPD-1 are seen in mice and humans with ARDS (Fig. 2a–d). The findings of a distinct protein and not just detection of membrane bound PD-1 in apoptotic bodies were further supported by the increased RNA expression of PD-1∆Ex3 (the alternative splice variant of PD-1 gene) in ARDS patients (Fig. 2e).

Experiments were done to assess the impact of sPD-1 on the immune system. In splenocytes from mice without ARDS there was reduced expression of TNF-α when exposed to BAL fluid with sPD-1 as compared to BAL fluid where sPD-1 been absorbed out (Fig. 3b). This speaks to the notion that the PD-1 system alters cytokine release/production, particularly TNF-α [25].

In ex vivo experiments, splenocytes derived from mice with ARDS or controls were used to further identify the role of sPD-1. PD-1 signaling has a greater influence on the production of cytokines than the regulation of proliferation [25]. We noted a transient increase, at 1 ng/mL sPD-1 treatment, in cytokine (Fig. 4b–e) release by cells derived from non-ARDS mice over the 0 ng sPD-1 treated group, no such change was seen in cells from ARDS mice vs. 0 ng sPD-1 group (Fig. 4b–e). This suggests splenocytes in ARDS may be an “exhausted cell” phenotype that has been reported in chronic viral infection [26]. Along these lines it’s worth noting that while increasing the concentration of sPD-1 (2.5–10 ng/mL sPD-1) in culture ablated the rise in cytokine release detected in 1 ng/mL sPD-1 treatment group, no effects were evident in cells derived from ARDS mice. (Fig.  4b–e). Interestingly, “exhaustion” could also pertain to IFN-γ as cells from mice with ARDS were not stimulated to produce this cytokine with the addition sPD-1. sPD-1 has been suggested as a treatment for “exhaustion” in chronic viral conditions, but recent work suggests stimulation of IFN-γ with sPD-1 may require the addition of another adjuvant molecule [27].

Animal models of ARDS are associated with increased levels of IL-6, MCP-1, and TNF-α [9, 19]. TNF-α is elevated and thought to be responsible for the pathologic changes that occur in ARDS [28–30]. BAL fluid from mice with ARDS reduces TNF-α production when sPD-1 is present. TNF-α is known to be directly influenced by PD-1 ligation [25]. In PD-1 deficient animal’s survival after ALI is improved and TNF-α production is drastically reduced in their lungs [9]. Here we suggest that sPD-1 ligation with PD-1 leads to a reduction in the TNF-α production, akin to the lack of TNF-α production seen in PD-1 deficient mice with ARDS. The capacity of sPD-1 in ARDS mouse BAL to decrease TNF-α level that we have shown here may in fact represent aspects of an endogenous mechanism directed at suppressing the inflammatory response associated ARDS in an attempt to dampen the immune system and improve outcomes.

T-cells from patients with aplastic anemia [11] and arthritis [23] that were cultured with recombinant sPD-1 lead to increased proliferation of the T-cells due to sPD-1 blocking PD-L1 [31]. In our experiment, culturing splenocytes with sPD-1 did result in increased proliferation, but this effect was comparable irrespective of whether or not the cells had been derived from animals with or without ARDS (Fig. 4a). When levels of protein were increased to 1000 ng/mL there was a decrease in proliferation compared to physiological levels (1–10 ng/mL, Fig. 2). These findings are consistent with data suggesting T cells and dendritic cells should be co-cultured [32] rather than purified T cells, as done in aplastic anemia and arthritis studies [11, 23, 31].

The resultant decline in proliferation seen at high concentrations of sPD-1 may be due to stimulation of PD-1 with sPD-1. PD-1 ligation is associated with decreased Bcl-xL [33] and its possible ligation of PD-1 by sPD-1 causes Bcl-xL levels to drop to where apoptosis predominates. Previous studies culturing T cells and dendritic cells with sPD-1 have suggested a similar explanation [32].

In mice and humans with ARDS sPD-1 is increased in both the serum and the BAL fluid; suggesting its value as a possible biomarker. In addition ROC curves predicted ARDS when looking at human and mouse BAL and mouse serum. In this study, we did not have matched pairs of blood and BAL samples to assess the impact of the combined levels in predicting ARDS. This potential use of sPD-1 as a biomarker must be studied further and include other clinical scores and biomarkers.

## Conclusion

We propose the release sPD-1 may attempt to dampen the immune response, as demonstrated by reduced cell proliferation and decreased cytokine production at physiological levels of sPD-1 and its presence could act as a potential biomarker in ARDS.

## Abbreviations

ARDS: acute respiratory distress syndrome
BAL: bronchial alveolar lavage
CLP: cecal ligation and puncture
fl: full length
PD-1: programmed cell death receptor-1
ROC: receiver operator characteristic
sPD-1: soluble programmed cell death receptor-1
